# Supplementary material for: Synergistic Activity of Remdesivir–Nirmatrelvir Combination on a SARS-CoV-2 In Vitro Model and a Case Report
Source: Viruses. 2023 Jul 19;15(7):1577. doi: 10.3390/v15071577 (PMC10385213; doi:10.3390/v15071577)
Supplement: Supplementary file 1 [file viruses-15-01577-s001.zip › viruses-2454524-Supplementary.pdf]

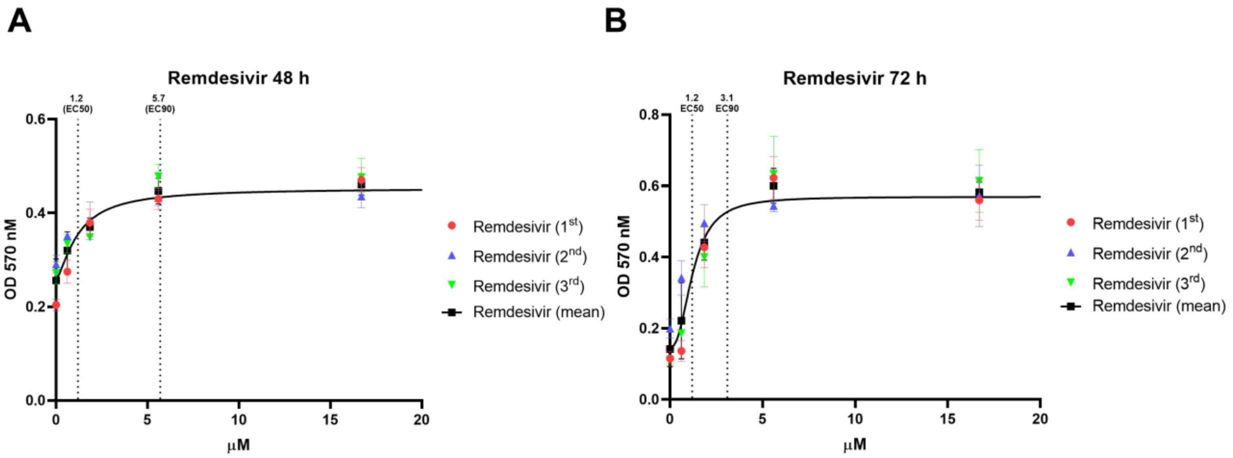

Supplementary Figure S1. Four-parameter variable-slope regression modeling of remdesivir. Vero E6 cells were infected with Severe Acute Respiratory Syndrome Coronavirus 2 (SARS-CoV-2) 20A.EU1 strain and treated with different concentrations of the compound for (**A**) 48 and (**B**) 72 h. The viability of cells was assessed by 3-[4,5-dimethylthiazol-2-yl]-2,5-diphenyl tetrazolium bromide (MTT) reduction assay and expressed as optical density (OD) 570nm ± SD. Data are the mean of at least 3 experiments with three technical replicates.

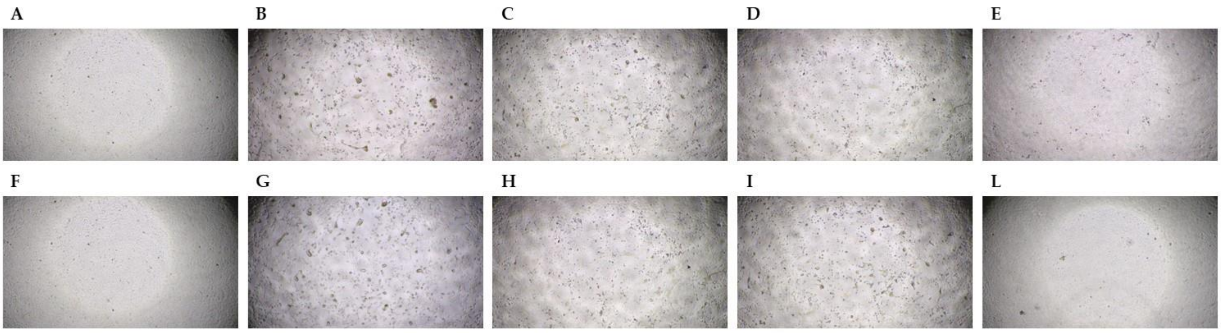

Supplementary Figure S2. Cytopathic effect (CPE) of Vero E6 cells infected with SARS-CoV-2 20A.EU1 strain incubated for 48 h (panel **A-E**) and 72 h (panel **F-L**). **A** and **F**: mock infected cells. **B** and **G**: untreated control. **C**: infected cells treated with nirmatrelvir 0.9 µM. **D**: infected cells treated with remdesivir 1.4 µM. **E**: infected cells treated with the combination nirmatrelvir 0.9 µM and remdesivir 1.4 µM. **H**: infected cells treated with nirmatrelvir 1.1 µM. **I**: infected cells treated with remdesivir 0.8 µM. L: infected cells treated with the combination nirmatrelvir 0.9 µM and remdesivir 1.4 µM. Pictures were taken with Optic Microscope Nikon Europe TS2 (magnification 40x)
